# CT characteristics of solitary pulmonary capillary hemangioma versus lung adenocarcinoma

**DOI:** 10.1186/s40644-025-00978-7

**Published:** 2026-01-10

**Authors:** Qi Wan, Jiaxuan Zhou, Xinyi Guo, Jilong Qin, Haiyan Xu, Qinru Wang, Weicong Chen, David F. Yankelevitz, Claudia I. Henschke, Xinchun Li, Yeqing Zhu

**Affiliations:** 1 Department of Radiology, The First Affiliated Hospital of Guangzhou Medical University, Guangzhou, 510120 China; 2 Department of Medical Technology, The Second People’s Hospital of Huizhou, Huizhou, 516001 China; 3 Department of Pathology, The First Affiliated Hospital of Guangzhou Medical University, Guangzhou, 510120 China; 4 The Department of Diagnostic, Molecular, and Interventional, Icahn School of Medicine at Mount Sinai, One Gustave L. Levy Place, New York, 10029 USA

**Keywords:** Solitary pulmonary capillary hemangioma, CT characteristics, Lung adenocarcinoma

## Abstract

**Background:**

Solitary pulmonary capillary hemangioma is an underrecognized rare benign disease that can radiologically mimic lung adenocarcinoma. This study aims to demonstrate radiologic features of SPCH and compare characteristics between SPCH and lung adenocarcinoma.

**Materials and methods:**

This retrospective study included all histologically confirmed SPCH cases from our institution between July 2015 and December 2024. For comparison, lung adenocarcinomas matched to SPCH by nodule consistency and size within the same period were included. Two chest radiologists independently assessed each nodule’s consistency, size, location, and morphologic signs, with group comparisons by χ² or Fisher exact tests and Mann-Whitney U tests.

**Results:**

The study comprised 44 SPCH patients (28 women, 63.6%; median age, 44.5 years [IQR, 37.0–55.2]) and 352 adenocarcinoma patients (202 women, 57.4%; median age, 58 years [IQR,50.0–65.0]). SPCH can manifest as nonsolid (12/44, 27.3%), part-solid (8/44, 18.2%) and solid nodules (24/44, 54.5%). SPCH shared multiple features with lung adenocarcinoma in nonsolid and part-solid nodules. However, in solid nodules, SPCH were more likely to occur in lower lobes (21/24, [87.5%] vs. 71/192 [37%]), more often presented with air bronchogram (18/24 [75.0%] vs. 26/192 [13.5%]), and lacked spiculation (2/24 [8.3%] vs. 169/192, [88.0%]) and pleural retraction (5/24, [20.8%] vs. 131/192, [68.2%]) (all *P* < 0.001). Five SPCH cases (5/44, 11.3%) demonstrated atypical bronchus lucency, while 3 cases (3/44, 6.8%) showed perivascular lucency.

**Conclusion:**

Solid SPCHs are distinguished from lung adenocarcinomas by a predilection for peripheral lower-lobe location, frequent air bronchograms, and near-absence of spiculation or pleural retraction. Recognition of atypical bronchus lucency and perivascular lucency signs may further aid differentiation.

**Supplementary Information:**

The online version contains supplementary material available at 10.1186/s40644-025-00978-7.

## Introduction

Solitary pulmonary capillary hemangioma (SPCH) is a rare type of benign tumor, first reported in 2006 by Fugo et al. [1]. Histologically, it is characterized by diffuse proliferation of capillary vessels in pulmonary alveolar septa and remains unclassified in the 2021 World Health Organization taxonomy of thoracic tumors [2]. Published data on SPCH are sparse and predominantly in the form of case reports focusing on pathology findings [3–7].

SPCH typically presents as an incidental solitary pulmonary nodule on computed tomography (CT), manifesting as nonsolid, part-solid or solid nodules, often raising a concern for malignancy, particularly lung adenocarcinoma. Accurate differentiation from more common neoplasms is therefore essential for guiding clinical management. To date, only one study has described detailed radiologic characteristics of SPCH, comprising 17 cases [8].

The purpose of this study was to investigate the clinical and radiologic characteristics of SPCH in a large cohort of histologically confirmed cases from our institution and compare these findings with those of lung adenocarcinoma.

## Materials and methods

### Participants

**Fig. 1:**
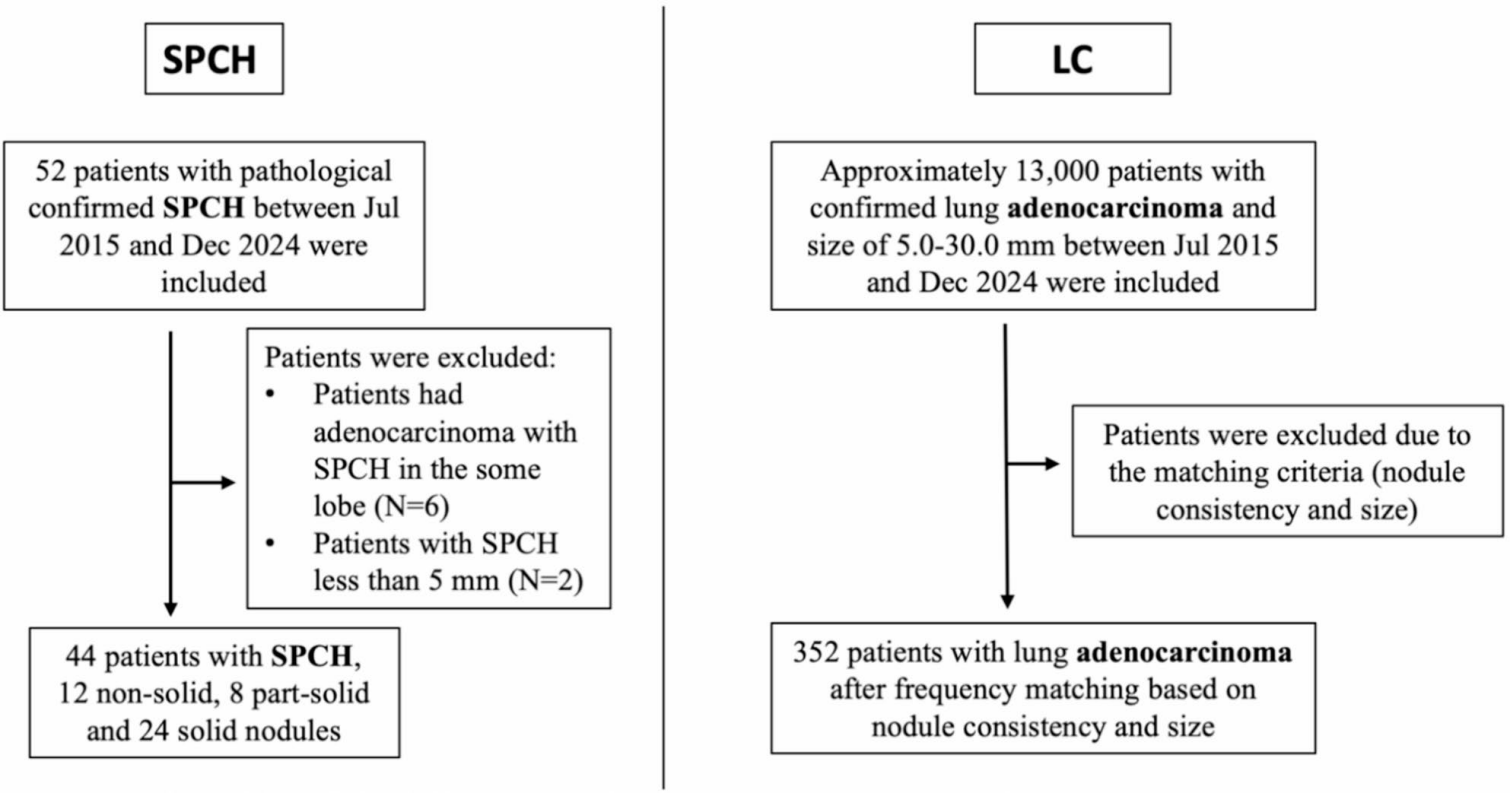
Flowchart for identification of 44 patients with SPCH and 352 patients with lung adenocarcinoma. Note.— LC = lung adenocarcinoma; SPCH = solitary pulmonary capillary hemangioma

This study was approved by our institutional review board and the requirement for informed consent was waived. We retrospectively reviewed all patients with pathological diagnoses of SPCH between July 2015 and December 2024 in our institution. The inclusion criteria were patients who underwent surgical resection had histologically confirmed diagnosis of SPCH, and had at least one chest CT scan in our institution before surgery. Patients were excluded according to the following criteria: (1) poor CT imaging quality due to obvious respiratory motion artifacts; (2) difficult to match the nodules on CT with pathological results for patients who had multiple nodules in the same lobe on CT images and pathological results revealed adenocarcinoma and SPCH; (3) nodules are too small (less than 5.0 mm in average diameter) to evaluate its features (Fig. 1). The pathological characteristics of 32 SPCH in our study were reported previously (6). For comparison, lung adenocarcinomas matched to SPCH based on nodule consistency (nonsolid, part-solid, solid) and size within the same period were included. Lung adenocarcinoma patients were extracted from our lung cancer registry.

### CT examinations

All CT scans were performed using standard-dose with or without contrast, with a slice thickness of 1 mm or less. All scans were performed on a 64-detector row or a 128-detector row CT scanner (SOMATOM Definition AS, Siemens Healthineers, Erlangen, Germany). The CT scan was obtained from the lung apices to the bases in a single breath-hold at maximum inspiration, with the following settings: tube voltage, 110–130 kVp; tube current, 100–140 mAs (using automatic current modulation technology); collimation, 0.5-1.25 mm; reconstruction slice thickness, 1 mm; interval, 1 mm; and image matrix, 512 × 512.

### Evaluation of radiologic characteristics of SPCH and lung adenocarcinoma on CT

All CT scans were reviewed by two senior chest radiologists (Q.W. and Y.Z., both with 13 years of experience) independently, blinded to patients’ clinical data and histological diagnosis. All lung adenocarcinoma cases were evaluated on non-contrast HRCT. SPCH cases were evaluated on HRCT, with a CECT subset available in 18/44 (40.9%) for enhancement assessment. Nodule characteristics were documented as following: consistency (solid, part-solid and nonsolid) [9, 10], size (average of the maximum longest dimension of the nodule and its width defined as the longest perpendicular to the length), location (peripheral: the distance to costal pleura was less than one-third of the total distance to hilum-costal pleura; central: the distance to costal pleura was more than one-third of the total distance to hilum-costal pleura) [11, 12], margin (clear or unclear), shape (regular [round or oval] or irregular), lobulation sign, reticulation sign, bubble-like lucency sign (spots of air attenuation or low density similar to normal lung tissue within nodule), pleural retraction sign [13], air bronchogram sign, perivascular lucency sign (circumferential low density around an intranodular vessel) [8], atypical bronchus lucency sign (irregular or angulated, bronchus-like lucency within the nodule without a continuous connection to a proximal airway) and enhancement pattern (hypodense, isodense, or hyperdense comparing to adjacent muscle).

After initial evaluation, any disagreement on the characteristics of each nodule was jointly reviewed by the two radiologists. If consensus was unattainable, a third chest radiologist (X.L. with over 20 years of experience) provided the final classification.

### Pathology analysis

Histologic diagnoses of SPCH were all made by surgical resection. One specialist pathologist (J.Q. with more than 15 years’ experience) reviewed all tissue samples carefully. SPCH was diagnosed based on pathologic features such as the proliferation of capillaries in the alveolar septa, and the vascular nature of SPCH was examined via immunohistochemistry using the vascular marker CD31 [1, 3].

### Literature search

To contextualise our findings, we performed a targeted PubMed search (January 1 2000 - March 31 2025) using the term “solitary pulmonary capillary haemangioma”. Titles and abstracts were screened and full texts were reviewed. Backward citation tracking was conducted. English-language case reports or series with histologic confirmation were included. No minimum sample size and no other exclusions, aside from duplicates or non-SPCH. Items including age, sex, lesion size, nodule consistency, location were extracted from literature.

### Statistical analysis

Continuous variables were summarized as means and standard deviations for normally distributed data or as medians and interquartile ranges for nonparametric data. Categorical variables were summarized as frequencies and percentages. Differences in features of SPCH and lung adenocarcinoma were compared by using the *x*^*2*^ test or Fisher exact test for categorical variables. The Mann-Whitney U was used for continuous variables. Two-sided *P* < 0.05 was considered to be a statistically significant difference. All analyses were performed with R software (version 3.6.3; R Foundation for Statistical Computing, Vienna, Austria).

## Results

### Participants

Between July 2015 and December 2024, a total of 52 consecutive patients with histologically confirmed SPCH were found in our institution. Eight cases were excluded due to their small size (less than 5 mm, *N* = 2) or mixed with other lung nodules in the same lobe (*N* = 6) (Fig. 1). Eventually, 44 SPCH with available CT images were included in this study. The cohort comprised 28 (63.6%) female and 16 (36.4%) male patients with a median age of 44.5 years (IQR, 37.0–55.2). The mean interval between the last CT examination and surgical resection was less than 20 days. Median nodule diameter was 9.2 mm (IQR, 7.4–12.0). The most common indication that led to the diagnosis of SPCH was screening for lung cancer (72.7%, 32/44) and physical checkup (27.3%, 12/44). Thirteen cases (5 nonsolid, 2 part-solid, and 6 solid) had follow-up scans with a median (range) follow-up of 13 (3-118) months. 92.3% (12/13) were unchanged in size and attenuation, while 1 case (nonsolid nodule) showed an increase in size in 10 years follow-up without density change. Reviewing the initial radiology reports, 68.1% (30/44, 10 nonsolid, 6 part-solid and 14 solid) of SPCH cases were misdiagnosed as adenocarcinoma in situ or lung adenocarcinoma, 11 (25%, 2 nonsolid, 2 part-solid, and 7 solid) were suggested as focal inflammation or inflammatory granuloma, 2 (4.5%, both solid) as sclerosing pneumocytoma, and 1 (2.3%, solid) as hamartoma (Table 1).

**Table 1:** Clinical demographics, nodule characteristics and radiologic diagnosis in SPCH

	SPCH, *n* (%)
**Gender**	
Female	28 (63.6)
Male	16 (36.4)
**Age**, median (IQR), y	44.5 (37.0-55.2)
**Nodule consistency**	
Non-Solid	12 (27.3)
Part-solid	8 (18.2)
Solid	24(54.5)
**Size**, median (IQR), mm	9.2 (7.4–12.0)
5.0–10.0 mm	26(59.1)
10.0–15.0 mm	14(31.8)
15.0–30.0 mm	5(9.1)
**Follow-up**	
No changes	12(27.3)
Increased	1(2.3)
Not available	31(70.5)
**Radiological diagnosis as per report**	
Adenocarcinoma in situ or lung adenocarcinoma	32 (72.7)
Infection	9 (20.5)
Pulmonary sclerosing pneumocytoma	2 (4.5)
Hamartoma	1 (2.3)

During the same period, there were 352 consecutive lung adenocarcinomas included in the final comparison analysis after matching based on nodule consistency and size (Fig. 1). Among the 352 lung adenocarcinoma patients, there were 202 (57.4%) female and 150 (42.6%) male patients with a median age of 58.0 years (IQR, 50.0–65.0) and a median nodule diameter of 10.0 mm (IQR, 7.5–14.5).

### Clinical demographics and nodule characteristics of *SPCH*

Clinical demographics and nodule characteristics in the overall SPCH cohort are shown in Table 1 and Table S1. All SPCH were solitary, with the majority located in the lower lobes (17 in right lower lobe [RLL] and 16 in left lower lobe [LLL]), followed by right upper lobe (RUL, *N* = 5), right middle lobe (RML, *N* = 5) and left upper lobe (LUL, *N* = 1). Most (35/44, 79.5%) of SPCH were located in the peripheral lung zone. For nodule consistency, 54.5% (24/44) of SPCH were solid nodules, 27.3% (12/44) were nonsolid, and 18.2% (8/44) were part-solid nodules. More than half of the SPCH had an average diameter ≤ 10.0 mm (26/44, 59.1%). Morphologically, 52.3% (23/44) SPCH had a regular shape, and 52.3% (23/44) had clear margins. A large majority of these nodules lacked spiculations (42/44, 85%) or pleural retraction (36/44, 82%). Air bronchogram was present in 25 of 44 SPCH cases (56.8%). Of the 18 cases with contrast-enhanced CT scans, 66.7% (12/18) were hypodense compared to adjacent muscle, while 22.2% (4/18) were isodense and 11.1% (2/18) were hyperdense.

### Comparison between SPCH and adenocarcinoma

**Fig. 2:**
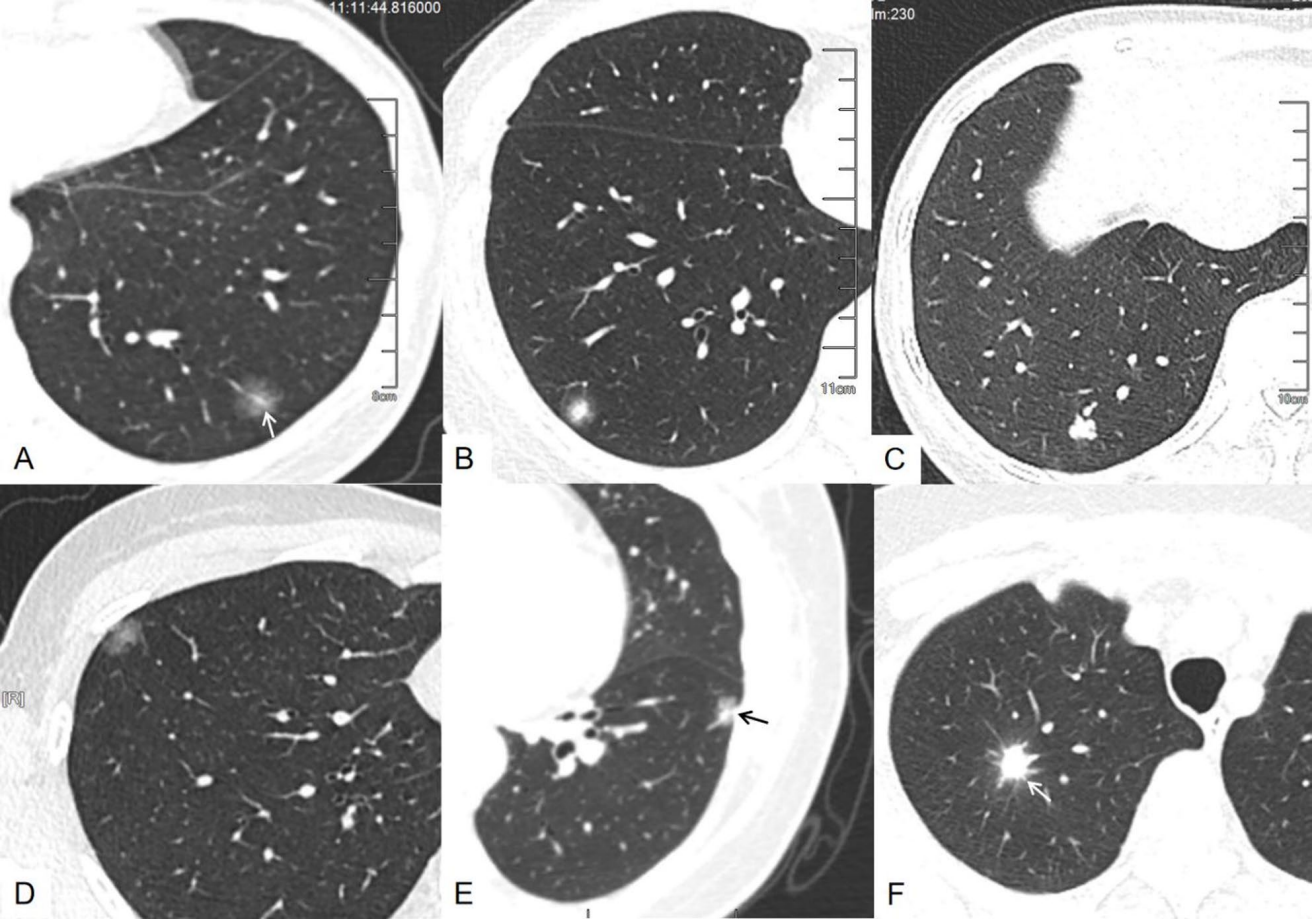
Matched exemples of solitary pulmonary capillary hemangioma (SPCH) and lung adenocarcinoma (LC) by consistency and size. **A**–**C**, SPCH; **D**–**F**, LC. **A**, 36-year-old woman: non-solid nodule in the left lower lobe with a traversing vessel (arrow). **B**, 33-year-old woman: part-solid nodule in the right lower lobe with unclear margin, no pleural retraction. **C**, 47-year-old woman: solid nodule in the right lower lobe with lobulation, no spiculation. **D**, 36-year-old woman: non-solid LC in the right upper lobe with clear margin and subtle pleural retraction. **E**, 51-year-old woman: part-solid LC in the left lower lobe with pleural retraction (arrow). **F**, 73-year-old woman: solid LC in the right upper lobe with lobulation and spiculation (arrow)

Comparison between clinical and radiologic features between SPCH (*N* = 44) and lung adenocarcinoma (*N* = 352) according to nodule consistency are detailed in Table 2 and illustrated in Fig. 2. In the nonsolid nodule group, only nodule location and margin were significantly different between SPCH and lung adenocarcinoma, with SPCH more common in the lower lobes (8/12, 66.7%) and had an unclear margin (7/12, 58.3%) while lung adenocarcinomas were more in the upper lobes (72/96, 75.0%) and had a clear margin (71/96, 74.0%) (both *p* < 0.001). In the part-solid nodule group, SPCH patients were younger than lung adenocarcinoma patients (46.1 years vs. 61.2 years, *p* < 0.001). The presence of spiculation were borderline significantly different between SPCH and lung adenocarcinoma (*p* = 0.047). In the solid group, age, location, margin, presence of air bronchogram, lobulation, spiculation and pleural retraction were significantly different in these two groups (all *p* < 0.001). Specifically, patients with SPCH were younger than those with lung adenocarcinoma (median 43.8 vs. 57.1 years, *p* < 0.001). SPCH were more likely to be located in the lower lobes (21/24, 87.5%), while lung adenocarcinomas were more commonly in upper lobes (121/192, 63.0%) (*p* < 0.05). SPCH were more likely to contain air bronchograms (18/24, 75.0%) and lacked lobulation (8/24, 33.3%), spiculation (2/24, 8.3%) and pleural retraction (5/24, 20.8%), while lung adenocarcinomas more frequently exhibited clear margins (162/192, 84.4%), lobulation (186/192, 96.9%), spiculation (169/192, 88.0%) and pleural retraction (131/192, 68.2%) (all *p* < 0.05).

**Table 2:** Demographic and CT characteristics of solitary pulmonary capillary hemangioma versus lung adenocarcinoma in non-solid, part-solid, and solid nodules

	Nonsolid nodule			Part-solid nodule			Solid nodule		
Variables	SPCH (*n* = 12)	Lung cancer (*n* = 96)	P-value	SPCH (*n* = 8)	Lung cancer (*n* = 64)	P-value	SPCH (*n* = 24)	Lung cancer (*n* = 192)	P-value
**Age**	51.7 ± 10.3	52.9 ± 10.7	0.718	46.1 ± 14.0	61.2 ± 8.1	< 0.001	43.8 ± 10.7	57.1 ± 10.9	< 0.001
**Size (average diameter**,** mm)**	9.6 ± 3.2	8.4 ± 2.7	0.154	9.7 ± 2.2	14.2 ± 7.4	0.092	9.9 ± 3.7	11.9 ± 4.6	0.053
**Gender**			0.778			0.540			0.596
Male	4 (33.3%)	36 (37.5%)		2 (25.0%)	23 (35.9%)		10 (41.7%)	91 (47.4%)	
Female	8 (66.7%)	60 (62.5%)		6 (75.0%)	41 (64.1%)		14 (58.3%)	101 (52.6%)	
**Location**			0.006			0.071			< 0.001
LUL	0 (0.0%)	29 (30.2%)		1 (12.5%)	9 (14.1%)		0 (0.0%)	38 (19.8%)	
LLL	2 (16.7%)	13 (13.5%)		0 (0.0%)	8 (12.5%)		14 (58.3%)	24 (12.5%)	
RUL	3 (25.0%)	38 (39.6%)		1 (12.5%)	30 (46.9%)		1 (4.2%)	68 (35.4%)	
RML	1 (8.3%)	5 (5.2%)		2 (25.0%)	4 (6.2%)		2 (8.3%)	15 (7.8%)	
RLL	6 (50.0%)	11 (11.5%)		4 (50.0%)	13 (20.3%)		7 (29.2%)	47 (24.5%)	
**Shape**			0.943			0.449			0.163
Round/oval/ polygonal	8 (66.7%)	63 (65.6%)		3 (37.5%)	16 (25.0%)		12 (50.0%)	68 (35.4%)	
Irregular	4 (33.3%)	33 (34.4%)		5 (62.5%)	48 (75.0%)		12 (50.0%)	124 (64.6%)	
**Margin**			0.021			0.651			0.009
Clear	5 (41.7%)	71 (74.0%)		3 (37.5%)	19 (29.7%)		15 (62.5%)	162 (84.4%)	
Unclear	7 (58.3%)	25 (26.0%)		5 (62.5%)	45 (70.3%)		9 (37.5%)	30 (15.6%)	
**Air bronchogram**			0.535			0.791			< 0.001
Absent	8 (66.7%)	72 (75.0%)		5 (62.5%)	43 (67.2%)		6 (25.0%)	166 (86.5%)	
Present	4 (33.3%)	24 (25.0%)		3 (37.5%)	21 (32.8%)		18 (75.0%)	26 (13.5%)	
**Lobulation sign**			0.100			0.065			< 0.001
Absent	8 (66.7%)	82 (85.4%)		6 (75.0%)	26 (40.6%)		8 (33.3%)	6 (3.1%)	
Present	4 (33.3%)	14 (14.6%)		2 (25.0%)	38 (59.4%)		16 (66.7%)	186 (96.9%)	
**Spiculation sign**			0.471			0.047			< 0.001
Absent	12 (100.0%)	92 (95.8%)		8 (100.0%)	42 (65.6%)		22 (91.7%)	23 (12.0%)	
Present	0 (0.0%)	4 (4.2%)		0 (0.0%)	22 (34.4%)		2 (8.3%)	169 (88.0%)	
**Pleural retraction sign**			0.067			0.439			< 0.001
Absent	11 (91.7%)	63 (65.6%)		6 (75.0%)	42 (65.6%)		19 (79.2%)	61 (31.8%)	
Present	1 (8.3%)	33 (34.4%)		2 (25.0%)	22 (34.4%)		5 (20.8%)	131 (68.2%)	
**Perivascular lucency sign**			NA			NA			< 0.001
**Absent**	12(100%)	96 (100%)		8 (100%)	64 (100%)		21(91.6%)	192 (100%)	
Present	0(0%)	0 (0%)		0 (0%)	0 (0%)		3(8.3%)	0 (0%)	
**Atypical bronchus lucency sign**			NA			0.25			< 0.001
Absent	12(100%)	96 (100%)		7 (87.5%)	64 (100%)		20 (83.3%)	192 (100%)	
Present	0(0%)	0 (0%)		1 (12.5%)	0 (0%)		4 (16.7%)	0 (0%)	

Note.— Categorical variables are presented as number of patients with percentages in parentheses and continuous variables are presented as mean with standard deviation

LC = lung adenocarcinoma; SPCH = solitary pulmonary capillary hemangioma

**Fig. 3:**
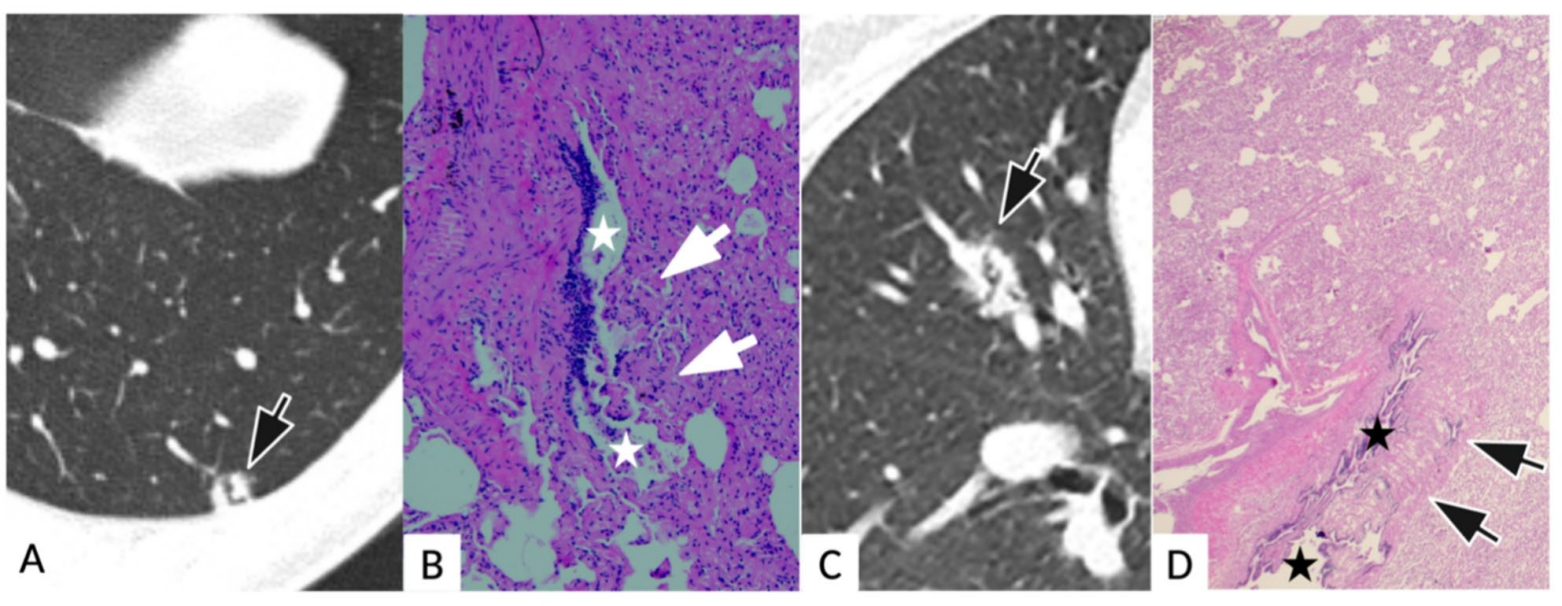
Radiological and pathological images of atypical bronchus lucency in two solitary pulmonary capillary hemangioma (SPCH) cases. Case 1, **A** and **B**. **A**: transverse CT images of a 40-year-man with a solid nodule in the left lower lobe. An irregular atypical bronchus lucency forming a right angle arises within the nodule (arrow) with a lumen diameter markedly larger than adjacent bronchioles; **B**: H&E images of the same patient showed irregular dilated bronchiole with capillary proliferation(arrows) protrusion into the bronchiolar lumens (stars), causing polyp-like projections. Case 2, C and D. **C**: transverse CT images of a 45-year-woman with a solid nodule in the right middle lobe. Within the lesion, the “antler-like” atypical bronchus lucency (arrow); **D**: H&E images of the same patient showed irregular dilated bronchiole with capillary protrusion (arrows) into the bronchiolar lumens(stars)

**Fig. 4:**
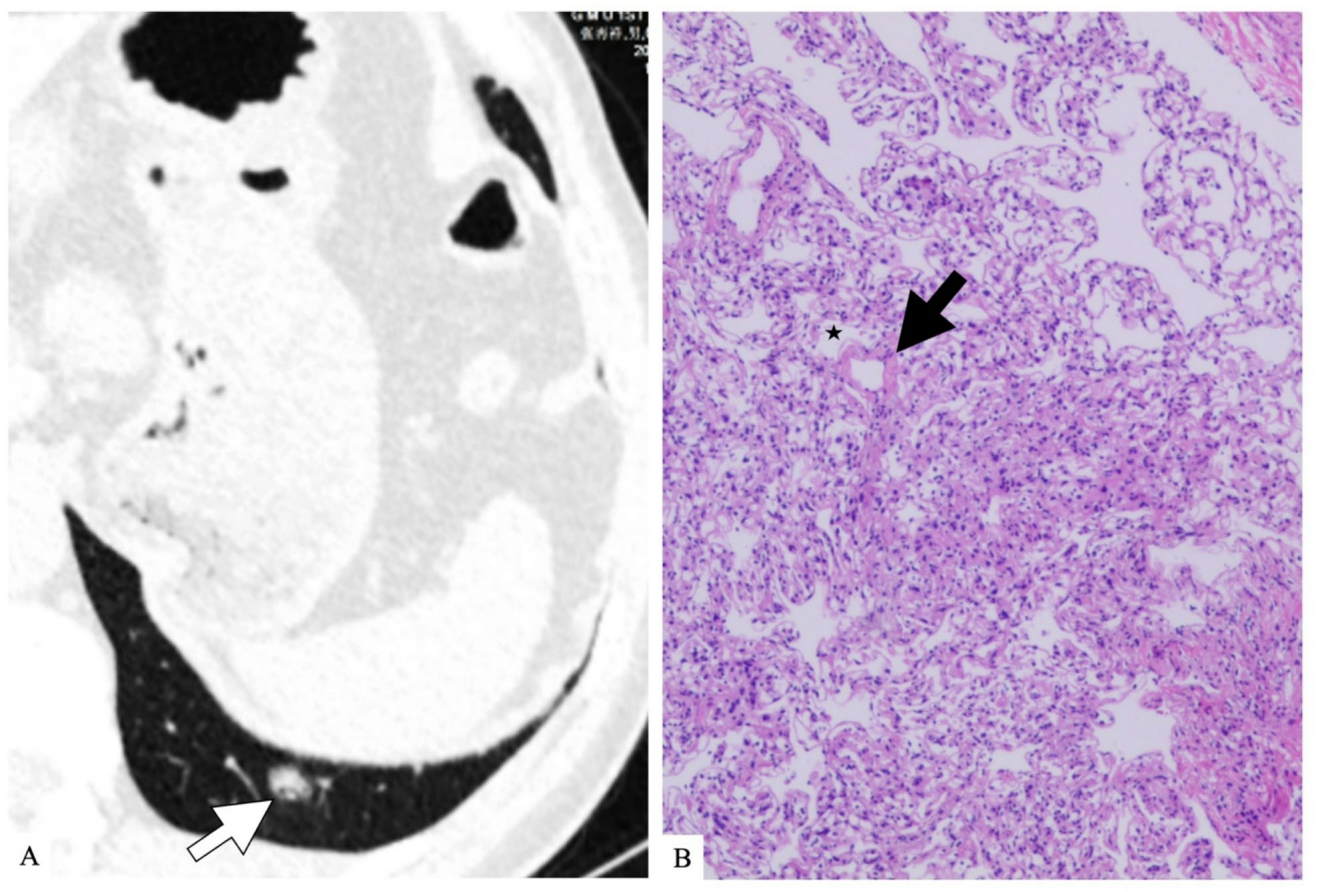
Example of radiological and pathological images of perivascular lucency in a solitary pulmonary capillary hemangioma (SPCH). **A**): transverse CT image of a 64-year-old woman who had a solid SPCH in the left lower lobe with perivascular lucency (arrow). **B**): pathological image of the same patient revealed a dilated pulmonary vein (arrow) with adjacent relatively spared capillary proliferation (star) in a solid SPCH

For special signs, atypical bronchus lucency occurred in solid (4/24, 16.7%) and part-solid SPCH (1/8, 12.5%) and corresponds pathologically to an irregular dilated bronchiole with capillary proliferation protrusion into the bronchiolar lumen (Fig. 3). Perivascular lucency was observed only in solid SPCH (3/24, 12.5%) (Fig. 4). On pathology, it demonstrated dilated pulmonary veins and edematous congestion in adjacent interlobular septa with relatively spared capillary proliferation. Pooled across consistency strata, atypical bronchus lucency was seen in 5/44 (11.4%) SPCH and 0/352 (0%) adenocarcinomas (*p* < 0.001), and perivascular lucency in 3/44 (6.8%) vs. 0/352 (0%) (*p* < 0.001).

### Summary of literature review

There were 17 studies [1, 3–5, 7, 8, 14–24] with a total of 76 cases of SPCH that have been reported to date in the English literature (Table 3). There were 44 (57.9%) females and 32 (42.1%) males with a mean age of 50, ranging from 26 to 68 years. SPCH were more common in the lower lobes (49/76, 64.5%), with the majority being part-solid (44/76, 57.9%), while 27.6% (21/76) were pure nonsolid nodules and 11.8% (9/76) solid and 2.6% (2/76) cystic. Of 39 cases with reported locations, 94.9% (37/39) were located in the peripheral lung zone while 5.1% (2/39) were in the central lung zone. One study [8] reported that the presence of air bronchograms was a common finding in SPCH (10 /17, 58.8%), while two cases demonstrated perivascular lucency (2/17, 11.8%).

**Table 3:** Summarization characteristics of previously reported SPCH in literature

First author and year	No. of SPCH	Age	Gender		Size	Nodule consistency					Lobe of nodule						Nodule location	
		(mean)	F	M	(mean)	Solid	Part-solid	Nonsolid	Cystic		RUL	RML	RLL	LUL	LLL		Peripheral	Central
fugo 2006	2	52	1	1	13	1	0	1	0		0	1	0	0	1		2	0
matsushita 2012	1	58	0	1	7	0	1	0	0		0	0	0	0	1		1	0
Lee 2012	2	53	0	2	7	2	0	0	0		0	1	0	1	0		2	0
Isaka 2013	1	55	1	0	6	0	1	0	0		0	0	0	0	1		1	0
Sakaguchi 2014	1	53	1	0	20	0	0	1	0		0	0	0	1	0		1	0
hashimoto 2016	7	54	4	3	11	0	6	1	0		0	2	3	0	2		n/a	n/a
hashimoto 2017	1	46	0	1	9	0	1	0	0		0	0	0	0	1		1	0
zhu 2017	1	40	1	0	18	0	0	1	0		1	0	0	0	0		1	0
zhao 2018	9	47	6	3	13	3	3	2	1		0	0	5	0	4		9	0
Hsieh 2018	16	49	11	5	8	0	6	9	1		1	2	5	5	3		n/a	n/a
hashimoto 2021	1	43	1	0	10	0	1	0	0		0	0	0	0	1		n/a	n/a
hashimoto 2020	1	49	0	1	7	0	1	0	0		0	0	0	0	1		1	0
Komatsu 2020	1	54	0	1	8	0	0	1	0		1	0	0	0	0		1	0
wang 2022	13	55	7	6	10	1	8	4	0		4	1	3	1	4		n/a	n/a
kim 2022	17	47	10	7	13	1	15	1	0		1	1	8	2	5		15	2
Kanamoto 2023	1	38	1	0	7	0	1	0	0		1	0	0	0	0		1	0
Hagui 2023	1	63	0	1	18	1	0	0	0		0	0	1	0	0		1	0
**Total**	**76**	**50**	**44**	**32**	**11**	**9**	**44**	**21**	**2**		**9**	**8**	**25**	**10**	**24**		**37**	**2**

Note.— F = Female; LLL = left lower lobe; LUL = left upper lobe; M = male; n/a = not available; RLL = right lower lobe; RML = right middle lobe; RUL = right upper lobe; SPCH = solitary pulmonary capillary hemangioma

## Discussion

In this study, we have presented the largest cohort of SPCH to date, comprising 44 histologically confirmed cases with CT scans from our institution. SPCH exhibited a female predominance and was frequently located in the lower lobes and peripheral lung zone. Over half of SPCH showed air bronchogram, while the majority lacked spiculation and pleural retraction signs.

Our results were consistent with the aggregate literature of 76 SPCH cases [1, 3–5, 7, 8, 14–24], which demonstrated that SPCH were more common in females (44/76, 57.9%), located in the lower lobes (49/76, 64.5%) and peripheral lung zone (37/39, 94.9%). Only a single study [8] in the literature has reported detailed CT characteristics of SPCH in a cohort of 17 cases. Kim et al. [8] revealed that most SPCH had smooth contours (16/17, 94.1%), air bronchogram (10/17, 58.8%) and ill-defined margins (9/17, 52.9%), while none had spiculation or irregular contour; perivascular lucency was seen in 2/17 (11.7%) and 14/15 (93.3%) demonstrated hypodense postcontrast enhancement. Our results corroborate these findings, showing similar characteristics including unclear margins(7/12, 58.3%), frequent air bronchograms (25/44, 56.8%), a lack of spiculations (2/44, 4.5%) or pleural retraction (8/44, 18.2%), with predominantly hypodense enhancement patterns (12/18, 66.7%) on contrast-enhanced CT. Additionally, perivascular lucency was noted in three cases (3/44, 6.8%).

In our study, the majority of SPCH cases were initially thought to be primary lung adenocarcinomas, similar to the Kim et al study [8]. Unlike previous work, we performed a size-matched, CT-based comparison within three nodule-consistency strata to explore whether discriminating features change with internal attenuation pattern. In nonsolid and part-solid lesions, morphology overlapped almost completely—apart from a lower-lobe preference and slightly more ill-defined margins for SPCH, making differentiation challenging in clinical practice. However, in solid group, age, location, margin, presence of air bronchogram, lobulation, spiculation and pleural retraction were significantly different in these two groups (all *p* < 0.001). Specifically, SPCH were more likely to present air bronchogram (18/24, 75.0%) and lacked lobulation (8/24, 33.3%), spiculation (2/24, 8.3%) and pleural retraction (5/24, 20.8%), while lung adenocarcinomas more frequently exhibited clear margins (162/192, 84.4%), lobulation (186/192, 96.9%), spiculation (169/192, 88.0%) and pleural retraction (131/192, 68.2%).

Notably, five SPCH (5/44, 11.3%) in our study showed atypical bronchus lucency, which was not previously reported and absent in lung adenocarcinoma. Histologically, it showed as luminal dilation and irregularity, which may stem from proliferating capillaries extending into small bronchioles. Three cases of the SPCH had perivascular lucency sign, which was also not found in lung adenocarcinoma. Microscopic examination of the perivascular lucency area revealed dilated pulmonary vein and edematous congestion in adjacent interlobular septa with relatively spared capillary proliferation, which is similar to Kim et al. study [8].

Although SPCH can also manifest as nonsolid and part-solid nodules, SPCHs were mainly found to be solid nodules (54.5%) in our study. However, in the literature, most of the reported SPCH were part-solid (44/76, 57.9%). The inconsistency may be due to the different definitions of part-solid and solid nodules. In our study, the diameter of the solid component relative to the diameter of the entire nodule is 80% or more, the nodule was classified as solid [25–27]. According to this criterion, some solid nodules with peripheral halo signs or few ground glass opacities were classified as part-solid nodules in the literature [3, 8]. There have been two reported SPCH in the literature [5, 7] that presented as cystic lesions, which we did not find in our study.

This study has several limitations. First, we did not correlate the CT imaging features with histologic findings from tissue samples. While previous studies have explored the pathological characteristics of SPCH, our analysis focused solely on defining its radiologic manifestations to assist radiologists in recognizing SPCH on CT images. Second, we did not perform comparative analyses between SPCH and other pulmonary diseases, such as squamous cell carcinoma, inflammatory pseudotumor, tuberculosis, and fungal infections. Since SPCH present with solid, part-solid and nonsolid consistency just as adenocarcinomas do and this was the most common cause for misdiagnosis, we believe this comparison was the most meaningful.

In conclusion, SPCH closely mimics adenocarcinoma in non-solid and part-solid nodules, but solid SPCH displays distinctive CT characteristics, including a high frequency of peripheral lower-lobe location, intranodular air bronchogram, and an absence of spiculation or pleural retraction. Awareness of these characteristics—supplemented by the atypical bronchus-lucency and perivascular-lucency signs—should prompt consideration of SPCH and help avert unnecessary surgical resection.

## Supplementary Information

Below is the link to the electronic supplementary material.

Supplementary Material 1

## Data Availability

The data are not publicly available due to privacy or ethical restrictions.
