# Supplementary material for: CT characteristics of solitary pulmonary capillary hemangioma versus lung adenocarcinoma
Source: Cancer Imaging. 2026 Jan 10;26:24. doi: 10.1186/s40644-025-00978-7 (PMC12882214; doi:10.1186/s40644-025-00978-7)
Supplement: Supplementary file 1 — Supplementary Material 1 [file 40644_2025_978_MOESM1_ESM.docx]

**Table S1. Radiological characteristics in overall SPCH cohort.**

|  | SPCH, n (%) |
| --- | --- |
| **Location** |  |
| Central | 9 (20.5) |
| Peripheral | 35 (79.5) |
| **Margin** |  |
| Clear | 23(52.3) |
| Unclear | 21(47.7) |
| **Shape** |  |
| Round/oval/polygonal | 23(52.3) |
| Irregular | 21(47.7) |
| **Lobulation sign** |  |
| Absent | 22(50.0) |
| Present | 22(50.0) |
| **Spiculation sign** |  |
| Absent | 42(95.5) |
| Present | 2(4.5) |
| **Pleural retraction sign** |  |
| Absent | 35(81.4) |
| Present | 8(18.6) |
| **Bubble-like lucency** |  |
| Absent | 39(88.6%) |
| Present | 5(11.4%) |
| **Air bronchogram** |  |
| Absent | 19(43.2) |
| Present | 25 (56.8) |
| **Perivascular lucency sign** |  |
| Absent | 41 (93.2) |
| Present | 3 (6.8) |
| **Atypical bronchus lucency sign** |  |
| Absent | 39 (88.6) |
| Present | 5 (11.4) |
| **Enhancement compared to muscle** |  |
| Hypodense | 12 (27.3) |
| Isodense | 4 (9.1) |
| Hyperdense | 2 (4.5) |
| Not available | 26(59.1) |
